# Subchronic low-dose 2,4-D exposure changed plasma acylcarnitine levels and induced gut microbiome perturbations in mice

**DOI:** 10.1038/s41598-019-40776-3

**Published:** 2019-03-13

**Authors:** Pengcheng Tu, Bei Gao, Liang Chi, Yunjia Lai, Xiaoming Bian, Hongyu Ru, Kun Lu

**Affiliations:** 1 Department of Environmental Sciences and Engineering, University of North Carolina at Chapel Hill, Chapel Hill, NC 27599 USA; 2 Department of Environmental Health Sciences, University of Georgia, Athens, GA 30602 USA

## Abstract

The gut microbiota critically confers various health benefits, whereas environmental chemicals can affect its constitution and functionality thereby increasing disease risk. In the present study, we aim to evaluate the toxic effects of a wildly-used herbicide 2,4-D (2,4-dichlorophenoxyacetic acid) on the gut microbiome and host using an occupationally relevant dose. A mouse model was used combined with metagenomic sequencing and metabolomic profiling to examine the alterations induced by subchronic low-dose 2,4-D exposure in fecal and plasma samples. The metagenomics results revealed a distinct gut microbial community with profound changes in diverse microbial pathways including urea degradation, amino acid and carbohydrate metabolism in 2,4-D-treated mice. Moreover, the metabolomics results revealed that the metabolic profiles in treatment group were differentiated from control group in both fecal and plasma samples. Toxic effects on the host of 2,4-D at an occupationally relevant dose were observed indicated by decreased acylcarnitine levels in plasma. These findings indicated that 2,4-D can cause toxicity and substantially impact the gut microbiome in mice at occupationally relevant doses, inferring that the relationship between environmental contaminants and microbiota is largely underestimated calling for more comprehensive consideration of the toxicity of occupational exposures.

## Introduction

2,4-D (2,4-dichlorophenoxyacetic acid) has been commercialized as a herbicide since more than 70 years ago, and it is still one of the most wildly-used herbicides in the world including the United States^1^. More than 1000 products that are currently commercially available in the U.S. contain 2,4-D as an active ingredient^2^. 2,4-D mimics indole-3-acetic acid (IAA), the main natural auxin, at molecular structure level resulting in uncontrolled growth, together with the induction of overproduction of reactive oxidative species (ROS) and lipid peroxidation, eventually leading to death in plants^3,4^. Generally, 2,4-D is quickly absorbed if ingested orally in mice and human, followed by rapid urinary excretion primarily as unchanged 2,4-D. It usually takes much longer for absorption with dermal exposure^5^. Proximal tubules of the kidney are responsible for the active excretion of 2,4-D, and dose-dependent toxicity ensues including adverse effects on the eye, kidney, thyroid, adrenals and testes or ovaries when the dose exceeds the renal clearance capacity^6^.

As one of the oldest and most successful herbicides, the toxic effects of 2,4-D to animals and human have been extensively studied although some results were inconsistent^7,8^. For oral administration, the lethal dose (LD_50_) of 2,4-D in mice is 138 mg/kg; in rats, depending on the chemical form LD_50_ values ranges from 639 mg/kg to 1646 mg/kg^2^. For dermal exposure, LD_50_ values ranges from 1829 mg/kg to higher than 2000 mg/kg in rabbits^6^. In addition, a subchronic NOEL (No Observable Effect Level) was established at 15 mg/kg body weight per day in rats and a subchronic NOAEL (No Observable Adverse Effect Level) was established at 15 mg/kg body weight per day in mice^6^. An ADI (Acceptable daily intake) of 0.01 mg/kg body weight was established based on the one-year toxicity study in dogs and two-year study in rats with the NOAEL of 1 mg/kg body weight per day using an uncertainty factor of 100^5^. 2,4-D generally degrades rapidly under most environmental conditions, with the half-life less than a week for most soil types; it also has rapid degradation in water under aerobic conditions^5^. Even with relatively low levels in the environment, human exposure to 2,4-D may occur through the direct contact with agricultural or residential areas after its application as a weed control agent.

Mounting evidence indicates that the gut microbiome is essential to host metabolism and physiology, and perturbations of the gut microbiome are associated with a number of human diseases^9^. It is well established that numerous environmental chemicals are able to induce unwanted alterations in the gut microbiome, for instance, heavy metals^10,11^, pesticides^12,13^, artificial sweeteners^14–16^, antibiotics^17,18^ and others^19^. More importantly, those environmentally-driven disturbance in the gut microbial community comprises an assemblage of microbial pathways and metabolites that may lead to adverse effects on the host. For example, 6-month saccharin exposure in mice enriched pro-inflammatory microbial pathways and metabolites in gut microbiome in concert with an elevation of inflammation mediators in mouse liver^14^. Likewise, diazinon-induced alterations in gut microbial pathways and metabolites regarding neurotransmitters might contribute to the neurotoxicity of diazinon^12^. As an environmental chemical that is extensively utilized on agriculture, home lawns and roadsides, it is imperative for the comprehensive examination of its possible harmful health effects including the impact on gut microbiome. However, the effects of 2,4-D on gut microbiome have not been properly investigated yet. Thus, we used a mouse model, which was utilized to study the microbiome-xenobiotics interactions in previous studies, to investigate the interactions between 2,4-D and the gut microbiome^10,12,19^. Furthermore, the dose used in this study for subchronic 2,4-D exposure is lower than the NOAEL reported in previous studies^5^, and is also occupationally relevant^20^. At this dose, we still observed strong toxicity on the host manifested by distinct plasma metabolite profiles, suggesting underestimated 2,4-D occupational toxicity.

The purpose of this study is to provide the first evidence regarding the impact on the host metabolic profiles and gut microbiome by subchronic low-dose 2,4-D exposure. To achieve this goal, we combined metagenomic and metabolomic approaches for the examination of 2,4-D induced alterations in mouse plasma and fecal samples. After 13-week 2,4-D exposure, a shifted gut microbial composition was revealed with a series of significantly differentiated species. Marked alterations of gut microbial gene repertoires were discovered, with enrichment of genes involved in urea degradation and perturbations in amino acid and carbohydrate metabolism, suggesting an altered functional role of the gut microbiome. In addition, metabolomic profiling of both fecal and plasma samples exhibited differentiated metabolic profiles between control and treatment groups, suggesting perturbations in host metabolism and gut microbial metabolic activities. These findings demonstrated that occupationally relevant exposure of 2,4-D is able to induce toxicity by altering the metabolic profiles in host plasma especially acylcarnitines; meanwhile, strong perturbations in gut microbiota were also induced with changes in its microbial composition, metabolic pathways and metabolites, suggesting that the interaction between environmental contaminants and microbiota is largely underestimated and providing new insights regarding mechanisms contributing to the toxicity of environmental agents.

## Results

### Diversity analysis and taxonomic characterization of 2,4-D perturbed gut microbiome

**Figure 1:**
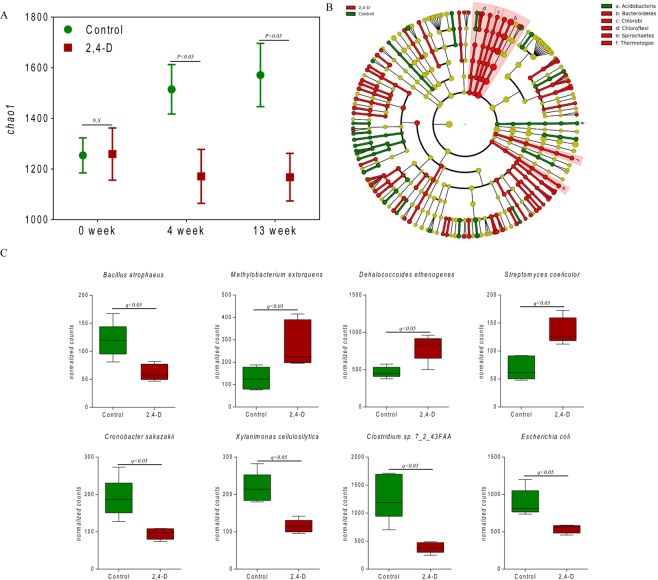
(**A**) Comparison of alpha diversities in the gut microbial communities between 2,4-D and control groups at 0 week, 4 weeks and 13 weeks. (student’s t-test, n = 5). (**B**) Cladogram for taxonomic representation of significant differences in the gut microbial communities at phylum level. (p < 0.05 (Kruksal-Wallis test); LDA score ≥2; n = 5). (**C**) Gut microbial species that are significantly different between control and 2,4-D groups. (q < 0.05; n = 5).

To investigate whether the mouse gut microbiome structure was disturbed by 2,4-D exposure, the gut microbial diversity and constitution were compared between treatment and control groups. Alpha diversities were measured and compared in the overall microbial community over the 13-week treatment course using the chao1 metric as shown in Fig. 1A. At 0 week, the alpha diversities showed no significant difference whereas the treatment group had significantly lower alpha diversities than control group at both 4 weeks and 13 weeks. The increase of alpha diversity in control group can be explained in view of the gut micro-ecosystem^21^. The chao1 index represents the richness of the gut microbial community. The richness of the gut microbiome would thrive in early life and gradually reach ecological saturation if not perturbed by intrinsic or extrinsic factors^22,23^, which accounts for the increase in alpha diversity in controls at 4 and 13 weeks. The reduction induced by 2,4-D exposure in alpha diversity indicates that the species richness, which was essential for the normal functional role of gut microbiota, was compromised by 2,4-D. Moreover, 13-week 2,4-D treatment substantially changed the gut microbial composition with diverse significantly-altered phyla and species. The phylum *Acidobacteria* was enriched in control group, whereas the phyla Bacteroidetes, *Chlorobi, Chloroflexi*, *Spirochaetes* and *Thermotogae* were enriched in treatment group (Fig. 1B). At the species level, the abundances of *Streptomyces coelicolor*, *methylobacterium extorquens* and *Dehalococcoides ethenogenes* were significantly higher in treatment group, whereas the abundances of *Clostridium sp. 7_2_43FAA*, *Xylanimonas cellulosilytica*, *Cronobacter sakazakii*, *Escherichia coli*, and *Bacillus atrophaeus* were significantly higher in control group (Fig. 1C). The alterations at both the phylum and species levels represented an interrupted microbial composition of the mouse gut microbiome after 2,4-D exposure.

### Comparative metagenome analysis revealed pronounced differences in gut microbial functional pathways

**Figure 2:**
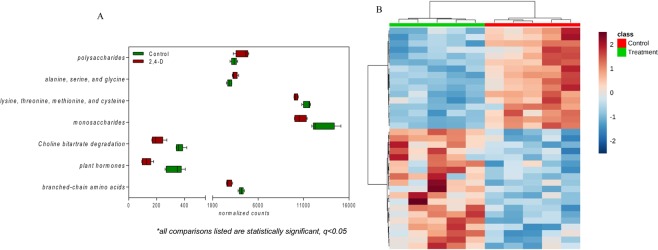
(**A**) Significantly-altered level 2 subsystems by 2,4-D exposure. (q < 0.05; n = 5). (**B**) Heatmap representing the distribution of gut microbial genes at level 3 subsystems indicating clear a clustering pattern within individual groups. (q < 0.05; n = 5).

The metagenomics RAST server^24^ (MG-RAST, version 4.03) was applied for the annotation of mouse gut metagenome to describe the metabolic features of the gut microbiome. SEED is a platform that provides consistent and accurate genome annotations^25^. SEED also includes subsystems, which stand for grouped biological functions together responsible for a specific biological pathway or structural complex^26^. The subsystems have 4 different levels based on classification levels. Figure 2 shows the gut microbial metabolic functions that were significantly changed by 13-week 2,4-D exposure. Of SEED subsystem level 2, the abundances of gut microbial genes involved in polysaccharides and alanine, serine, and glycine subsystems were significantly higher in treatment group, whereas the abundances of gut microbial genes involved in lysine, threonine, methionine, and cysteine, monosaccharides, choline bitartrate degradation, plant hormones and branched-chain amino acids subsystems were significantly higher in control group (Fig. 2A). Furthermore, the heat map representing the distribution of gut microbial genes of SEED subsystem level 3 displayed a consistent clustering pattern among individuals in separate groups (Fig. 2B). Taken together, the marked distinction in microbial metabolic pathways demonstrates that 2,4-D exposure did alter the functionality of gut microbiome.

**Figure 3:**
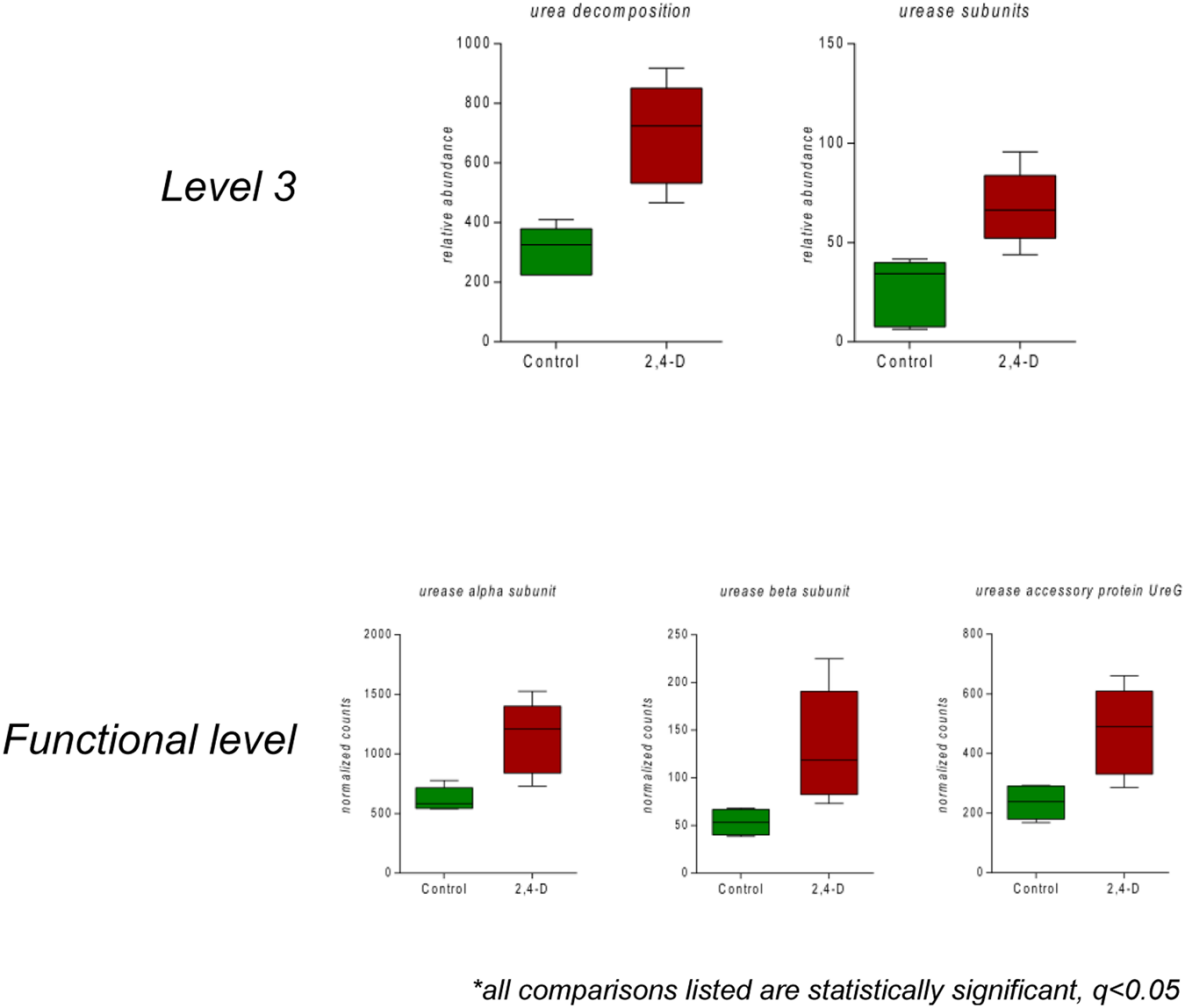
Comparison of microbial genes involved in urea degradation at level 3 subsystems and functional level subsystems in control and 2,4-D groups. (all comparisons listed are statistically significant, q < 0.05; n = 5).

**Figure 4:**
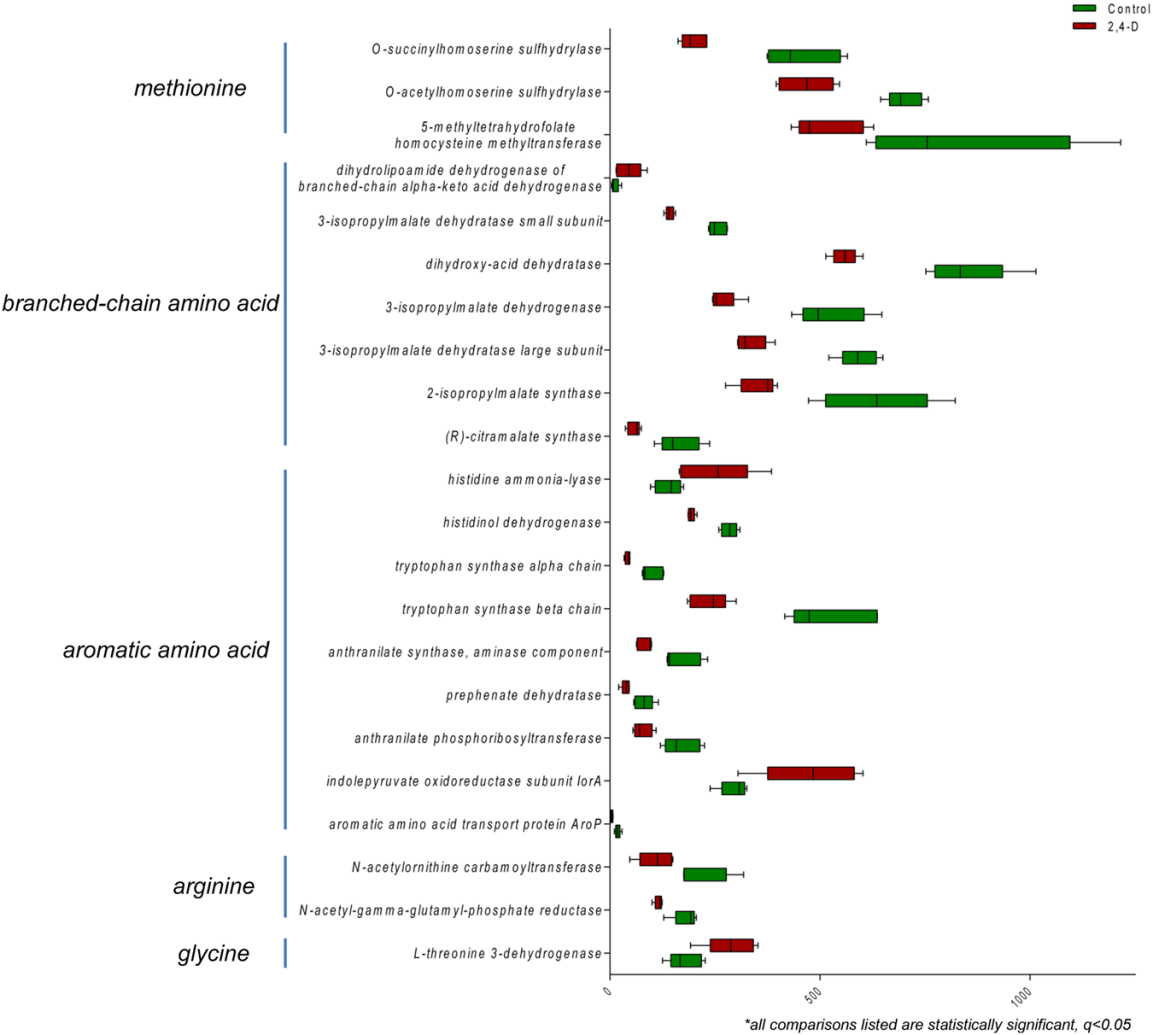
Comparison of microbial genes involved in amino acid metabolism in control and 2,4-D groups. (all comparisons listed are statistically significant, q < 0.05; n = 5).

**Figure 5:**
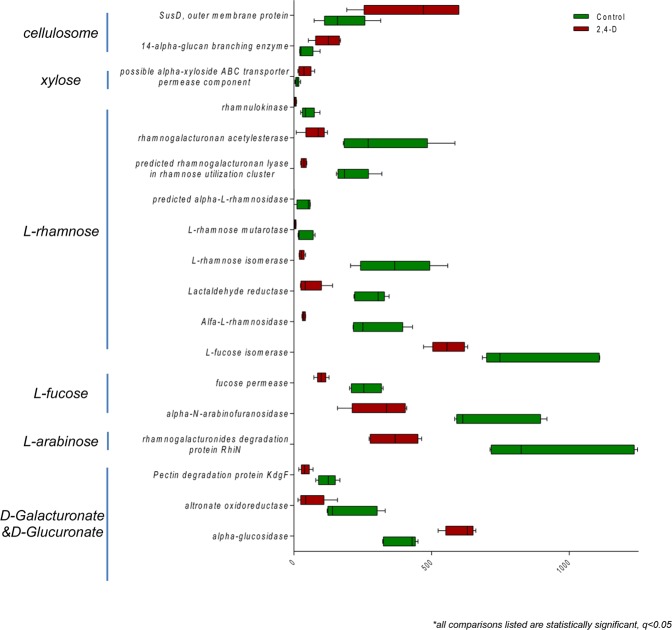
Comparison of microbial genes involved in polysaccharide and monosaccharide utilization in control and 2,4-D groups. (all comparisons listed are statistically significant, q < 0.05; n = 5).

Specifically, the gut microbiome of the treatment group exhibited significant alterations to pathways involved in urea degradation, amino acid metabolism and carbohydrate utilization compared to control group (Figs 3, 4 and 5). The pathway abundances of urea decomposition and urease subunits were significantly increased in 2,4-D treated mouse gut microbiome. Moreover, at functional level, the microbial genes of urease alpha subunit, urease beta subunit and urease accessory protein *UreG* were significantly upregulated (Fig. 3). In addition, significant perturbations in microbial amino acid metabolism were found in treatment group compared to control group with significantly-altered gene abundance involved in the metabolism of glycine, arginine, methionine, aromatic amino acids and branched-chain amino acids (Fig. 4). Significant alterations in the microbial genes involved in carbohydrate utilization were also observed in mouse gut microbiome of the treatment group compared to control group. In particular, the utilization of monosaccharides (e.g. L-arabinose, L-fucose and L-rhamnose) decreased whereas the utilization of polysaccharides (e.g. cellulosome) increased supported by the downregulation and upregulation of a repertoire of microbial genes encoding related enzymes, respectively (Fig. 5).

### Alterations in the metabolic profiles of gut microbiome induced by 2,4-D exposure

**Figure 6:**
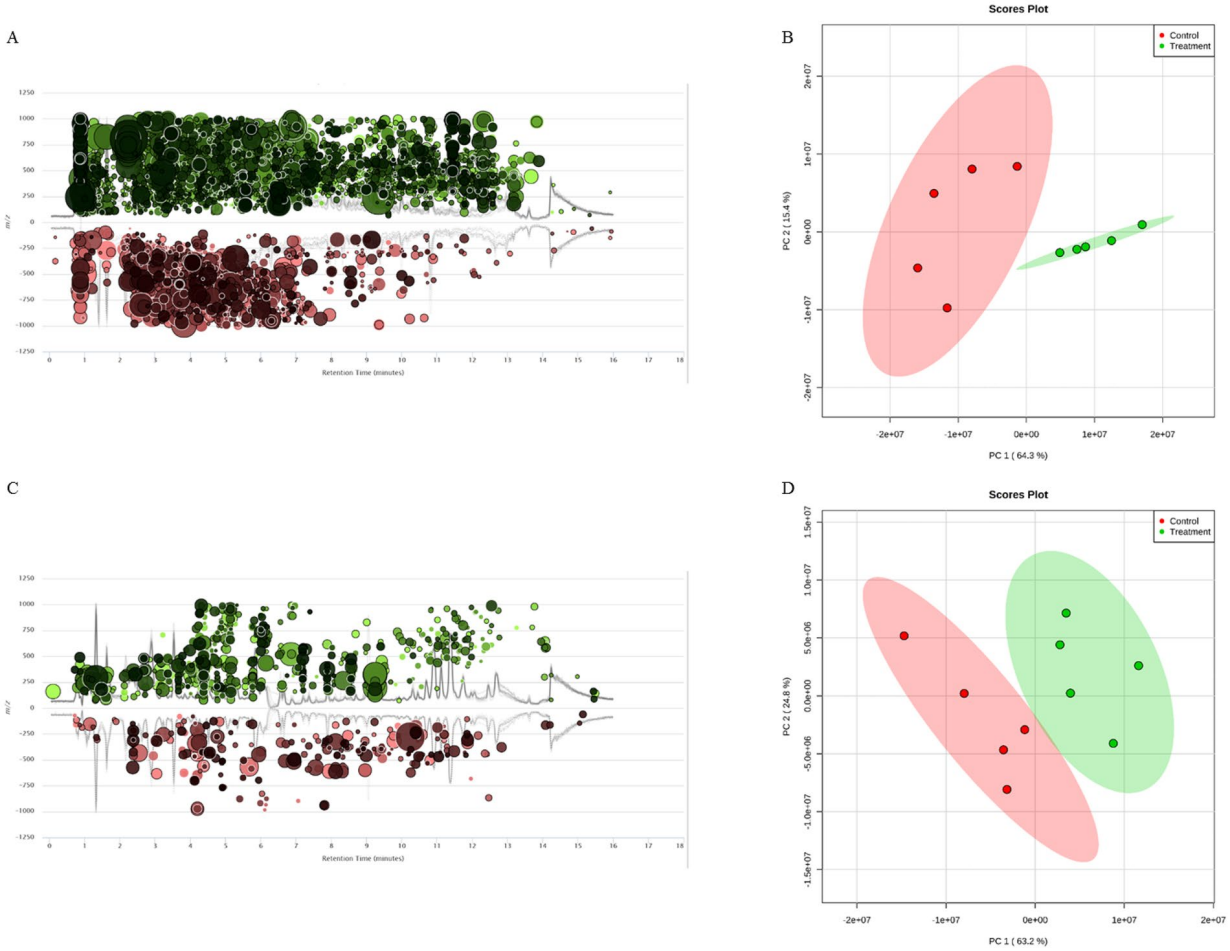
(**A**,**C**) Cloud plots representing 2,4-D exposure-perturbed metabolic profiles with molecular features being altered in mouse fecal and plasma samples, respectively. (n = 5). (**B**,**D**) Separations of the metabolite profiles between control and 2,4-D groups in mouse fecal and plasma samples, respectively. (n = 5).

To assess the impact of a shifted gut microbiome on its metabolic products, metabolomic profiling was performed on mouse fecal samples. Figure 6A illustrated that 2,4-D exposure changed the metabolic profiles of the mouse gut microbiome, with 6394 perturbed molecular features. Moreover, the metabolic profiles of treatment group were well-separated from control group using the first two components of PCA. (Fig. 6B, 64.3% and 15.4% variation were explained by PC1 and PC2, respectively). Together the data indicated that 2,4-D induced functional changes in the gut microbiome metabolic profiles. To further investigate the effects of the changes in the gut microbial metabolome, we identified 21 fecal metabolites that were significantly altered after 2,4-D exposure (Table S2). For instance, prostaglandins including delta-12-Prostaglandin J2 and prostaglandin-c2 were significantly more abundant in 2,4-D treatment mouse. In addition, nitrogen metabolites such as amino acids and their metabolic intermediates; purines, pyrimidines and their derivatives were also found to be differentiated, which supported that 2,4-D perturbed the nitrogen metabolism of mouse gut microbiome indicated by metagenomics results.

### Changes in host plasma metabolome after 2,4-D exposure

Host metabolomic profiles were assessed using mouse plasma samples. After 13-week 2,4-D exposure, the metabolic profiles were differentiated in mouse plasma between control and treatment groups. PCA analysis shows that the metabolic profiles of the two groups were almost separated (Fig. 6D, 63.2% and 24.8% variation were explained by PC1 and PC2, respectively). Furthermore, 1074 molecular features were perturbed in 2,4-D-treated mice compared to controls (Fig. 6C). We identified 17 perturbed metabolites in the plasma (Table S1). Of interest, the levels of a class of metabolites known as acylcarnitines were significantly lower in mice with 2,4-D treatment (Table 1). The distinct metabolic profiles in the plasma of 2,4-D-treated mice especially with a series of lower-level acylcarnitines indicated the toxic effects of subchronic 2,4-D exposure at an occupationally relevant dose.

**Table 1:** Plasma acylcarnitines that were significantly decreased in 2,4-D treated mice compared to controls.

Identified metabolites	Fold Change	p-value	M/Z	Retention Time (min)
cis-5-Tetradecenoylcarnitine	−1.7	0.031	370.2952	9.4
2-Hydroxylauroylcarnitine	−1.5	0.041	360.2739	6.7
Decanoylcarnitine	−1.7	0.006	316.2481	7.9
2-Hydroxymyristoylcarnitine	−1.6	0.01	388.3057	8.9
Dodecanoylcarnitine	−1.6	0.015	344.2794	9
Tetradecanoylcarnitine	−1.5	0.018	394.295	9.2

(Welch’s t test; n = 5; p < 0.05; fold change >1.5).

## Discussion

The mechanisms of 2,4-D toxicity in animals and human are not quite definite and oxidative stress may be one of them^27–30^. In the present study, metagenomic sequencing and metabolomic profiling were applied to examine the impact of subchronic low-dose 2,4-D exposure on the host and gut microbiome. Clearly, the host metabolic profiles were differentiated by 2,4-D exposure indicating low-dose toxicity. Meanwhile, the results also demonstrate that 2,4-D perturbed not only the composition and diversity, but also functional pathways and metabolic profiles of the gut microbiota. Accumulating evidence suggests that the alterations of microbiome-related pathways and metabolites would lead to a disturbed gut-host homeostasis hence increasing disease risk^18^. For example, perturbations in gut microbial metabolic activities involved in protein fermentation and amino acid metabolism can lead to the elevation of toxic metabolites such as polyamines, hydrogen sulphide and ammonia, which play an role in the progression of colorectal cancer by inducing inflammation, ROS production, or genotoxicity^31^. Likewise, microbiome-derived uremic toxins such as p-cresol sulfate and indoxyl sulfate resulting from gut dysbiosis are associated with chronic kidney disease^32,33^. Given the strong association between gut microbiome perturbations and a variety of human diseases coupled with the capacity of 2,4-D to induce gut microbiome alterations, the findings of the present study might provide insights regarding the mechanistic basis by which 2,4-D adversely affects human health.

One of the intriguing findings from our study was that 2,4-D induced remarkable abundance changes in gut microbial pathways and gene families. We found 35 microbial pathways (Level 3 Subsystems) and 82 microbial genes (Functional Level Subsystems) at significantly different abundances in the treatment group compared to control group. A reduction of genes involved in plant hormones was observed in mouse gut microbiome after 2,4-D exposure (Fig. 2A). Even though plant hormone-related pathways do not exist in animals and human, they are commonly present in gut bacteria. Thus, the alterations to bacterial metabolic activities and products resulting from the impact of 2,4-D on plant hormone metabolism may indirectly affect host health, which contradicts the notion that certain environmental chemicals are safe to human just because their targeting pathways are not found in the human body. We also discovered a consistent enrichment of pathways and genes involved in urea degradation in treatment group compared to control group (Fig. 3). It is estimated that almost up to one third of urea produced endogenously by human body is degraded by microbial urease^34^. More importantly, microbial urease has been recognized as a virulence factor for gastrointestinal pathogenesis^35^. In addition, a significant shift in gene abundances of microbial amino acid metabolism as well as carbohydrate metabolism occurred in the gut microbiome of 2,4-D treated mice. To be more specific, for amino acids, the metabolism of alanine, serine, and glycine was enhanced whereas lysine, threonine, methionine, cysteine and branched-chain amino acids was diminished; for carbohydrates, the metabolism of polysaccharides was enhanced whereas monosaccharides was diminished. Together this may indicate the change of utilization preference in amino acids and carbohydrates in the 2,4-D-altered gut microbial community, and evidence shows that microbial amino acids and carbohydrate metabolism can influence host amino acid and energy homeostasis, respectively^36,37^. Therefore, the fundamental metabolism and the functional role of the gut microbiome were different in 2,4-D-treated mice compared to controls due to changes in diverse microbial metabolic pathways and functions.

A less resilient gut microbial community indicated by a lower alpha diversity along with a variety of shifted gut microbial species was found in mice treated with 2,4-D (Fig. 1). The relative abundances of *Dehalococcoides ethenogenes* increased in the gut microbiome of treatment group compared to control group. *Dehalococcoides ethenogenes* has been shown to play a major role in the degradation of chlorinated hydrocarbons in contaminated environments^38,39^. It is suggested that *Dehalococcoides ethenogenes strain 195* has the dehalogenation potential to a diversity of chlorinated hydrocarbons based on up to 17 putative dehalogenase gene homologues found in its genome^40^. Thus, the enrichment of *Dehalococcoides ethenogenes* in 2,4-D-treated mouse gut microbiome likely reflects the selective pressure exerted by 2,4-D in the gut environment, and the competitive advantage held by *Dehalococcoides ethenogenes* resulting from its dehalogenation ability. In addition, an increased bacterial population in Phylum *Spirochaetes* has been found to be associated with 2,4-D exposure. Many members from Phylum *Spirochaetes* are involved in prevalent pathogenic diseases, such as *Brachyspira pilosicoli* and *Brachyspira aalborgi* in human intestinal spirochaetosis^41^, and *Leptospira* genus in Leptospirosis^42^. Various *Spirochaetes* species are also associated with the development of dementia and could be involved in the pathogenesis of Alzheimer’s disease^43^.

The communication between the gut microbiota and host through production of gut metabolites is one essential aspect of the gut-host cross-talk. On one hand, the gut microbial profiles are under regulations of host-produced metabolites, for instance, antimicrobial peptides^44^. On the other hand, gut microbiome-derived metabolites can contribute to the rise of either host fitness or disease risk^45^. For example, the gut microbiome is an important source for vitamins such as vitamin K, vitamin B12, biotin, folate and so forth^22,46^. Meanwhile, trimethylamine N-oxide, derived from the gut bacterial metabolite trimethylamine, is highly correlated with cardiovascular disease and kidney disease^47^. In the present study, we discovered distinct gut metabolic profiles between treatment and control groups, demonstrating the capacity of 2.4-D to alter the metabolic functions and metabolite fingerprints in the gut microbiome. Prostaglandins were found to be enriched in fecal samples after 2,4-D exposure in mice. Prostaglandins are a class of lipid autacoids involved in inflammatory response^48^, which can be found in intestinal mucosa^49^. It is speculated that the increase of prostaglandins might be one of the coping strategies with gut inflammation. However, the mechanisms by which the abundances of prostaglandins being elevated and the physiological effects on host are unknown and merit further studies. We also found significant perturbations in nitrogen metabolites in the gut microbiome. In concert with the alterations in gene abundances of amino acid metabolism, differentiated metabolites including several amino acids and their metabolic intermediates supported that 2,4-D treatment induced functional alterations in gut microbial amino acid metabolism. Additionally, the differences in purines, pyrimidines and their derivatives also indicated a perturbed nitrogen metabolism. Taken together, the alterations in the metabolite profiles of mouse fecal samples testify to 2,4-D-induced functional changes in the mouse gut microbiome.

Furthermore, the metabolite profiles in host plasma were differentiated by 13-week low-dose 2,4-D treatment, suggesting the capability of low-dose 2,4-D exposure to directly or indirectly impact host metabolism. In particular, the plasma levels of a series of acylcarnitines were observed to be significantly lower in 2,4-D-treated mice. The NOAEL for subchronic (13-week) exposure of 2,4-D in mice is 15 mg/kg body weight per day according to previous studies^5^, which is equivalent to 90 ppm 2,4-D in drinking water using the daily water intake as 8 ml per 30 g body weight^50^. The dose used in the present study was approximately 60 times lower. However, strong toxic effects of 2,4-D manifested by marked perturbations in the host plasma acylcarnitine levels were observed at this very low dose, indicating that the 2,4-D toxicity can be exerted at doses that are far lower than NOAEL. In this context, acylcarnitines have never been associated with 2,4-D exposure and/or 2,4-D toxicity before. Acylcarnitines are involved in the beta-oxidation of fatty acids^51^, and the alterations in the plasma levels of acylcarnitines suggested perturbations in the fatty acid beta-oxidation pathway. It is reported that the daily doses of 2,4-D exposure in professional turf applicators were predicted to be up to 20 mg per day, which is comparable to the dose in the present study^20^. Thus, monitoring the changes of plasma acylcarnitine levels could help to identify occupational toxicity of 2,4-D. In addition, growing evidence suggests an important role that acylcarnitines play in neurological diseases^52^. For instance, a decrease of serum acylcarnitines is reported to be associated with neurological disorders including Parkinson’s disease and Alzheimer’s disease^53,54^. Thus, lower levels of plasma acylcarnitines observed in 2,4-D-treated mice may be associated with the potential neurotoxicity of 2,4-D. Although the link between 2,4-D-induced gut microbiome perturbations and low plasma acylcarnitines cannot be built based on the results of the present study, clear correlations could be identified between the perturbed gut microbial species, *Xylanimonas cellulosilytica* and plasma acylcarnitine levels (Fig. S1). The association between low-dose 2,4-D exposure-induced gut microbiome perturbations and lower plasma acylcarnitine levels provided novel insights upon the mechanisms for 2,4-D toxicity, and the plasma acylcarnitines can serve as a novel biomarker for 2,4-D toxicity at low doses.

Despite the relatively limited sample size in this study, the results clearly demonstrated the capacity of 2,4-D to induce both phylogenetic and functional changes in mouse gut microbiome with differentiated metabolic profiles. Significant distinction was also shown in host plasma metabolic profiles indicating perturbations in host metabolism, even though direct causal effects of 2,4-D-disturbed gut microbiome on host metabolic outcomes cannot be inferred from the present study. Ours is the first study to investigate the impact on the gut microbiome by 2,4-D exposure using multi-omics approaches, the interplay between the alterations in microbial pathways and metabolites and their resultant health effects on host warrants further investigation.

To conclude, this is the first study that demonstrated the alterations in the gut microbiome driven by subchronic low-dose 2,4-D exposure. The metagenomics indicated significant perturbations by 2,4-D to the gut microbial composition and functionality, in concert with distinct metabolic profiles in mouse fecal samples revealed by metabolomics. Meanwhile, the metabolomic profiling of host plasma samples revealed altered host metabolic profiles suggesting 2,4-D toxicity at occupationally relevant doses. These findings support the notion that the homeostasis of gut microbiome can be readily impacted by environmental chemicals, suggesting reconsideration for the health effects and toxicity of wildly-used chemicals including 2,4-D.

## Methods

### Animals and exposure

Specific-pathogen-free (SPF) C57BL/6 male mice (~8 weeks of age), purchased from Jackson Laboratories (Bar Harbor, ME), were housed in the animal facility of University of Georgia. The environmental conditions were maintained as 22 °C temperature, 40–70% humidity, and a 12:12 hour light:dark cycle. All experiments were approved by the University of Georgia Institutional Animal Care and Use Committee. All methods were performed in accordance with the relevant guidelines and regulations. Water and standard pelleted rodent diet ad libitum were provided for 1 week for their acclimation before 2,4-D treatment. After 1 week of acclimation, a control (n = 5) and treatment (n = 5) group were then randomly assigned. For the 2,4-D exposure, 1ppm 2,4-D (Sigma-Aldrich, MO) water solution (the concentration used in the present study is lower than NOAEL, i.e., 15 mg/kg of body weight per day^5^) was administered to the mice in the treatment group replacing tap water while the mice in the control group received tap water over the course of 13 weeks. Regular monitoring for health conditions was done twice a week. The mice were treated humanely and with regard for alleviation of suffering. Before sacrifice, each mouse was placed in an individual cage for fecal sample collection, and the feces were immediately put into liquid nitrogen for snap freezing. Fecal samples were collected individually for both groups at the time points including 0 week, 4 weeks and 13 weeks; plasma samples were collected during necropsy; and the samples were kept at −80 °C after harvest until further analysis.

### 16S rRNA gene sequencing and diversity analysis

16S rRNA gene sequencing was performed as previously described in Lu *et al*.^10^. Briefly, microbial DNA was extracted from mouse fecal pellets using the PowerSoil® DNA isolation kit (MoBio Laboratories, CA) according to manufacturer’s instructions. Then the DNA was amplified using 515 (5′-GTGCCAGCMGCCGCGGTAA) and 806 (5′-GGACTACHVGGGTWTCTAAT) primers^55^ targeting the V4 regions of 16S rRNA gene in bacteria. After normalization, individual samples were barcoded and finally pooled for the construction of the sequencing library. The quantification of DNA was performed using the Qubit 2.0 Fluorometer (Invitrogen, Life Technologies), and the DNA was sequenced using the Illumina MiSeq (500 cycles v2 kit) in the Georgia Genomics Facility of University of Georgia. Paired reads were then assembled using Geneious 8.15 (Biomatters, Auckland, New Zealand). Operational taxonomic unit (OTU) picking and diversity analysis were conducted using Quantitative Insights into Microbial Ecology (QIIME, version 1.9.1).

### Comparative metagenomic analysis

Shotgun metagenomic sequencing was performed as previously described in Gao *et al*.^12^. Briefly, fecal DNA (10 ng/µL) was fragmented using the Bioruptor UCD-300 sonication device. The Kapa Hyper Prep Kit (Kapa Biosystems) was applied to construct the sequencing library according to manufacturer’s instructions. The quantification of DNA was performed using the Qubit 2.0 Fluorometer (Invitrogen, Life Technologies). The sequencing was performed using the Illumina NextSeq High Output Flow Cell (300 Cycles; PE150) in the Georgia Genomics Facility of University of Georgia. The MG-RAST metagenomics analysis sever (http://metagenomics.anl.gov, version 4.0.3) was applied for the taxonomic and functional analysis with RefSeq and Subsystems databases, respectively^24^.

### Sample processing for metabolomics

For fecal samples, 20 mg feces and 50 mg glass beads (Sigma-Aldrich, MO) were added to 400 µL cooled methanol solution (methanol: water 1: 1), followed by homogenizing using a TissueLyser (QIAGEN) for 15 min at 50 Hz. The supernatant was collected after centrifuging for 10 min at 1,2000 rpm, dried up in a SpeedVac (Thermo Scientific), and then resuspended for metabolomic profiling. For plasma samples, 80 µL cooled methanol was added to 20 µL plasma. After incubation for 30 min at −20 °C, the samples were centrifuged for 10 min at 1,2000 rpm. The supernatant was collected, dried up in a SpeedVac (Thermo Scientific), and then resuspended.

### Metabolomic profiling and metabolite identification

LC-MS analysis was performed on a quadrupole-time-of-flight (Q-TOF) 6550 mass spectrometer (Aglient Technologies, Santa Clara, CA) with an electrospray ionization source. The mass spectrometer was interfaced with an Agilent 1290 Infinity II UPLC system (Aglient Technologies, Santa Clara, CA). Metabolites were analyzed in the positive mode over a m/z range of 50–1000 with a C18 T3 reverse-phased column (Waters Corporation, Milford, MA). MS/MS data were generated on the Q-TOF for the identification of perturbed metabolites. The XCMS Online sever (https://xcmsonline.scripps.edu, version 3.5.1) was applied for peak picking, alignment, integration, and extraction of the peak intensities. A two-tailed Welch’s t-test was used for the assessment of metabolite differences between control and treatment groups. The programs of MS-DIAL^56^ (version 2.70) and MS-FINDER^57^ (version 2.20) were used for the identification of metabolites.

### Statistical analysis of data

Alpha rarefaction was performed using chao1 metric via QIIME. Also, cladogram for taxonomic representation of significant differences was generated using LefSe^58^. Principle component analysis (PCA) was applied to examine the intrinsic clusters of metabolomics data. In addition, heatmaps were generated using a hierarchical clustering algorithm to visualize the comparison of gene abundances. The metagenomic sequence count data for both taxonomic and functional analysis were processed using DESeq2^59^ for statistics analysis, and the result was considered to be significant if q-value < 0.05 (adjusted for the false discovery rate).

### Ethics approval

The animal experiment was approved by the University of Georgia Institutional Animal Care and Use Committee.

## Supplementary information

Supplementary Material

## Data Availability

Data is available through http://www.ebi.ac.uk/ena/data/view/PRJEB21490 with project accession #PRJEB21490.
