# Supplementary material for: Subchronic low-dose 2,4-D exposure changed plasma acylcarnitine levels and induced gut microbiome perturbations in mice
Source: Sci Rep. 2019 Mar 13;9:4363. doi: 10.1038/s41598-019-40776-3 (PMC6416245; doi:10.1038/s41598-019-40776-3)

***Supplementary Material***

**Subchronic low-dose 2,4-D exposure changed plasma acylcarnitine levels and induced gut microbiome perturbations in mice**

Pengcheng Tu^1^, Bei Gao^2^, Liang Chi^1^, Yunjia Lai^1^, Xiaoming Bian^2^, Hongyu Ru^1^,

and Kun Lu^1^*

1. Department of Environmental Sciences and Engineering,

University of North Carolina at Chapel Hill, Chapel Hill, NC, 27519

2. Department of Environmental Health Sciences,

University of Georgia, Athens, GA, 30602

* Corresponding Author

Kun Lu, PhD

Department of Environmental Sciences and Engineering

University of North Carolina at Chapel Hill, Chapel Hill, NC, 27599

Tel: 919-966-7337

Email: [kunlu@unc.edu](mailto:kunlu@unc.edu)

**Supplementary Tables**

| Table S1. Significantly-altered serum metabolites in 2,4-D-treated mice compared to controls. | | | | |
| --- | --- | --- | --- | --- |
| Identified metabolites | Fold Change | p-value | M/Z | Retention Time (min) |
| cis-5-Tetradecenoylcarnitine | -1.7 | 0.031 | 370.2952 | 9.4 |
| 2-Hydroxylauroylcarnitine | -1.5 | 0.041 | 360.2739 | 6.7 |
| Decanoylcarnitine | -1.7 | 0.006 | 316.2481 | 7.9 |
| 2-Hydroxymyristoylcarnitine | -1.6 | 0.010 | 388.3057 | 8.9 |
| Dodecanoylcarnitine | -1.6 | 0.015 | 344.2794 | 9.0 |
| Tetradecanoylcarnitine | -1.5 | 0.018 | 394.2950 | 9.2 |
| Cysteamine | 2.5 | 0.048 | 78.0419 | 4.4 |
| 3-Hydroxy-cis-5-tetradecenoylcarnitine | -2.2 | 0.030 | 386.2900 | 8.5 |
| 3-Hydroxy-9-hexadecenoylcarnitine | -1.8 | 0.035 | 414.3212 | 9.3 |
| 3-Hydroxy-5, 8-tetradecadiencarnitine | -1.5 | 0.044 | 384.2742 | 8.0 |
| 3-Hydroxy-9Z-octadecenoylcarnitine | -1.7 | 0.009 | 442.3527 | 10.2 |
| trans-2-Dodecenoylcarnitine | -2.4 | 0.001 | 342.2637 | 8.4 |
| 2-trans,4-cis-Decadienoylcarnitine | -1.5 | 0.040 | 312.2165 | 7.2 |
| 9-Hexadecenoylcarnitine | -1.8 | 0.006 | 398.3264 | 10.3 |
| Oleoylcarnitine | -1.6 | 0.012 | 426.3577 | 11.1 |
| 3, 5-Tetradecadiencarnitine | -1.6 | 0.013 | 368.2794 | 8.9 |
| L-Agaritine | -1.9 | 0.033 | 306.0803 | 1.3 |
| Guanosine | -1.8 | 0.005 | 284.0989 | 2.4 |
| Guanine | -1.8 | 0.005 | 152.0566 | 2.4 |
| dUMP | -1.5 | 0.023 | 309.0437 | 2.4 |
| L-Tyrosine | 2.1 | 0.042 | 182.0810 | 1.7 |

| Table S2. Significantly-altered fecal metabolites in 2,4-D-treated mice compared to controls. | | | | |
| --- | --- | --- | --- | --- |
| Identified metabolites | Fold Change | p-value | M/Z | Retention Time (min) |
| 4-(2-Amino-3-hydroxyphenyl)-2,4-dioxobutanoic acid | 2.5 | 0.005 | 224.0544 | 2.4 |
| 2-Aminomuconic acid semialdehyde | -1.6 | 0.001 | 142.0499 | 1.2 |
| Creatine | 2.7 | 0.009 | 132.0710 | 2.6 |
| Cholesterol sulfate | 3.5 | 0.050 | 467.3149 | 8.8 |
| Chromone | 1.6 | 0.002 | 147.0470 | 2.4 |
| 6-Ketoestriol | 3.8 | 0.013 | 325.1413 | 3.7 |
| L-Glutamine | 2.0 | 0.003 | 147.0764 | 1.0 |
| L-Arginine | -1.6 | 0.006 | 175.1137 | 2.2 |
| 12,13-DHOME | 1.9 | 0.008 | 337.2351 | 9.6 |
| S-Methylmethionine | 1.8 | 0.005 | 164.0730 | 2.4 |
| Delta-12-Prostaglandin J2 | 1.8 | 0.002 | 335.2194 | 10.4 |
| Prostaglandin-c2 | 1.7 | 0.005 | 335.2192 | 9.9 |
| Guanine | 2.0 | 0.006 | 152.0567 | 1.4 |
| Deoxycytidine | 1.6 | 0.008 | 228.0977 | 1.4 |
| Cytosine | 1.8 | 0.009 | 112.0507 | 1.5 |
| Alpha-ketoisovaleric acid | 2.3 | 0.002 | 117.0549 | 1.7 |
| 13'-Hydroxy-alpha-tocotrienol | 2.1 | 0.003 | 441.3368 | 12.6 |

**Supplementary Figures**

Figure S1. Scatter plots illustrating statictical association between the relative abundance of gut microbial species, *Xylanimonas cellulosilytica* and the mass spectrum intensities of plasma acylcarnitines. (blue: control, red: 2,4-D; rho>0.5; p<0.05)


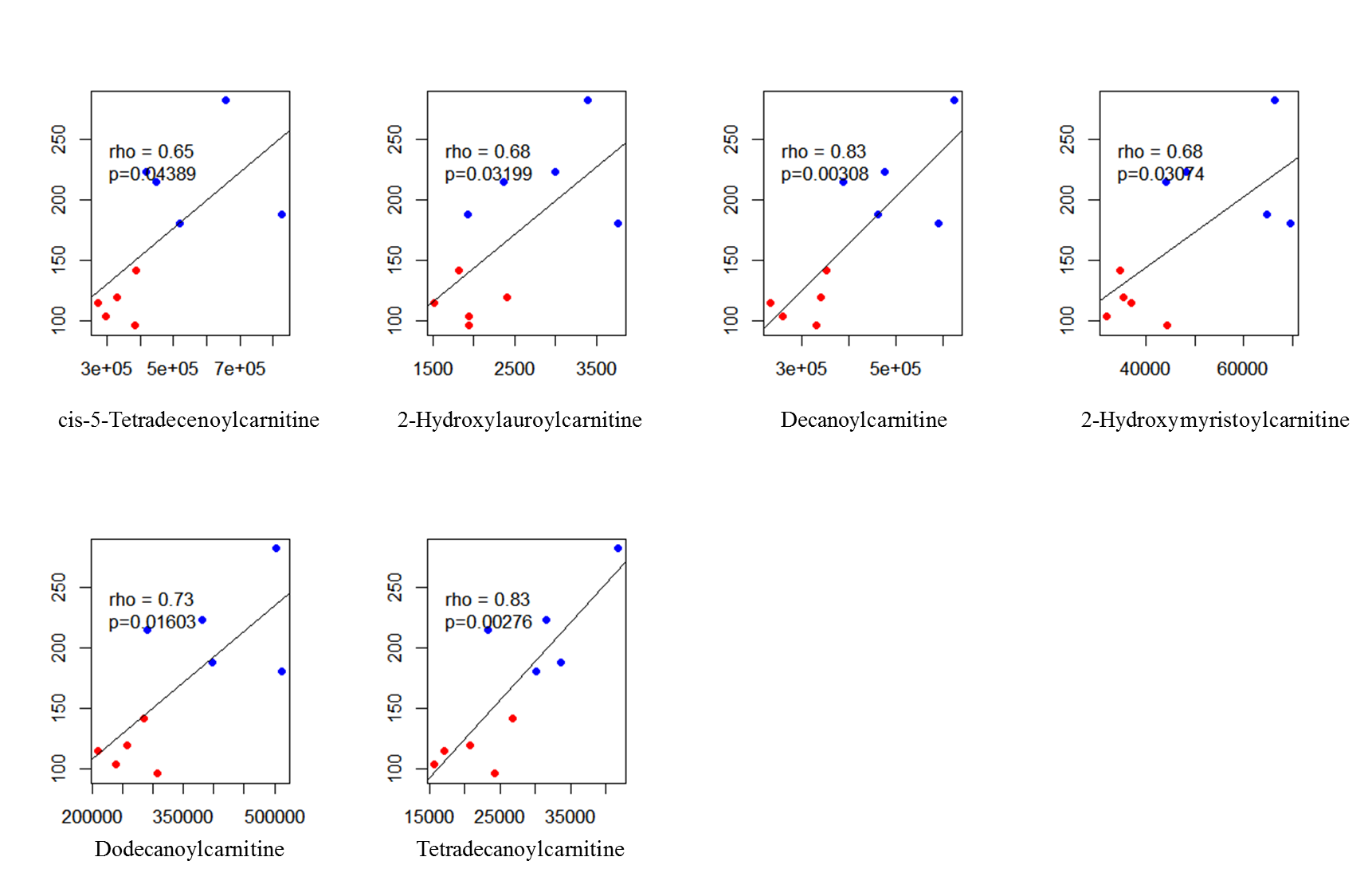

Supplement: Supplementary file 1 — Supplementary Material [file 41598_2019_40776_MOESM1_ESM.docx]
